# The Current State of FLT3 Inhibition in Acute Myeloid Leukemia – Pitfalls and Promises

**DOI:** 10.4172/2576-1471.1000166

**Published:** 2017-10-16

**Authors:** Katherine A Minson, Deborah DeRyckere, Douglas K Graham

**Affiliations:** 1 Aflac Cancer and Blood Disorders Center, Children’s Healthcare of Atlanta and Emory University, USA; 2 Department of Pediatrics, Atlanta, GA, USA

## Commentary

The development of novel targeted agents aimed at selective inhibition of dysregulated oncogenic pathways has been a major focus and advancing area in translational oncology research. In acute myeloid leukemia (AML) the first successes have been in targeting mutations in the receptor tyrosine kinase FLT3. Small molecule inhibitors targeting FLT3 have been in clinical use for the past two decades with many patients showing favorable initial responses; however, development of resistance occurs almost universally. Here we describe mechanisms of resistance to FLT3 inhibitors and ongoing studies aimed at overcoming it.

Mutations in FLT3 (FMS-like tyrosine kinase 3) are the most common genetic alteration in patients with AML occurring in approximately 30% of adult and 15% of pediatric patients at the time of diagnosis [1–3]. Primarily mutations consist of internal tandem duplications (ITD) of the juxtamembrane domain leading to constitutive receptor activation [4]; however, in 5-7% of patients activating lesions present at diagnosis are due to point mutations in the kinase domain [5,6] and mutations are also less commonly observed in the juxtamembrane domain [7–9]. The presence of an ITD mutation confers a poorer prognosis, particularly in the pediatric population. In one study, overall survival decreased from 44% in patients without a mutation to 7% for those with a FLT3-ITD mutation [3]. The prognostic significance of point mutations is less well defined [2,3,6]. Interestingly, while these mutations result in constitutive activation of the FLT3 receptor, the downstream effects are distinct from those observed following ligand-stimulation of wild-type FLT3 (FLT3-WT) [10,11]. FLT3-WT is normally expressed in hematopoietic progenitors and promotes proliferation and survival through activation of the downstream RAS/MEK/ERK and PI3K/AKT pathways. In contrast, ITD mutations contribute to leukemogenesis by preferentially inducing activation of STAT5 resulting in aberrant cell growth [10,11] and transcriptional repression of C/EBPα and PU.1, which mediate a block in myeloid differentiation [10–13].

Preclinical studies demonstrating robust anti-leukemic effects of FLT3 inhibition lead to development of ATP-competitive tyrosine kinase inhibitors (TKIs) targeting mutated FLT3 for clinical use. First generation agents with activity against FLT3 such as sunitinib, sorafenib, and midostaurin were multi-kinase inhibitors that also targeted related receptors such as PDGFR and KIT. Given the multi-kinase nature of these compounds, their use was limited due to poor potency against FLT3 and increased toxicity due to off-target activity. To address these concerns, the second-generation TKI, quizartinib (AC220) was developed with increased potency against and selectivity for FLT3. Initial responses to treatment with single-agent quizartinib were promising with 44% of relapsed or refractory FLT3-ITD AML patients achieving a composite complete remission in a phase II study [14]; however, responses were not durable and the impact on survival was limited with a median duration of response of 11 weeks indicating rapid development of resistance. Clinical use of quizartinib has also been limited by its off-target inhibition of c-KIT which has led to unacceptable myelosuppression [15].

Further analysis of patient samples to better understand mechanisms of relapse revealed secondary point mutations in the FLT3 kinase domain in patients who relapsed during quizartinib monotherapy.

The most common quizartinib-resistance conferring mutations occur at the D835 and F691 loci and confer cross-resistance to the first-generation inhibitor sorafenib [16–19]. Mutations at D835 occur in the FLT3 activation loop and serve to stabilize the protein in the active “DFG-in” conformation thereby preventing binding of type 2 TKIs such as quizartinib and sorafenib [16]. F691 is in the ATP-binding pocket of FLT3 and is a conserved gatekeeper residue. Similar mutations, such as T790M in EGFR [20] and T315I in BCR-ABL [21], have been well described as a mechanism of TKI resistance and substitute of a larger residue for a smaller one, thereby preventing binding of the inhibitor. Interestingly, while the D835 and adjacent I836 loci are the predominant site for FLT3 activation loop mutations in TKI-naïve AML [5,6], F691 mutations have not been described in the absence of the selective pressure of aninhibitor. The presence of a primary mutation at these sites is relevant as they confer the same differential sensitivity to FLT3 TKIs as the secondary mutations [22]. In the rare cases of point mutations in the juxtamembrane domain, sensitivity to inhibitors has not been well-studied but in one reported case sorafenib mediated initial but not sustained anti-leukemic effects in the presence of a L576Q mutation [9].

Development of secondary point mutations represent the best characterized mechanism of acquired resistance to FLT3 inhibition; however, resistant FLT3-ITD cells lacking secondary point mutations have been frequently identified [23] indicating that other mechanisms such as protection in the bone marrow niche and/or activation of bypass signaling pathways may account for the majority of cases. In the clinic, it has been anecdotally noted that FLT3 inhibitors induce a much more rapid clearance of leukemic blasts from the peripheral blood than from the bone marrow [24] leading to studies aimed towards identifying stromal-derived mediators of resistance. One consistent finding has been persistent activation of ERK in response to FLT3 inhibition in the presence of bone marrow stroma [24,25]. Although the specific mediators of this persistent activation are not fully understood, it has been proposed to occur through upregulation of the ligands fibroblast growth factor 2 (FGF2) and FLT3 ligand (FL). FL is upregulated in patients in response to chemotherapy [26] and the addition of exogenous FL or FGF2 blocks ERK inhibiton in response to quizartinib in AML cell lines [24]. As previously noted, the ERK pathway is preferentially regulated by FLT3-WT compared to mutated FLT3 and due to the characteristic block in myeloid differentiation in AML blasts, FLT3-WT is aberrantly expressed in the majority of patient samples and is often co-expressed with the mutant allele. Aberrant expression of FLT3-WT may therefore be responsible for ligand-mediated signaling and protection even in the presence of a FLT3 inhibitor. Indeed, the FLT3 inhibitors quizartinib and sorafenib preferentially inhibit FLT3-ITD over FLT3-WT and their efficacy is abrogated upon co-expression of the two molecules [27]. This mechanism may also explain the observation that FLT3-ITD allelic burden is a positive predictor of cytotoxicity in response to FLT3 inhibition [28]. These data outline a mechanism of resistance to FLT3 inhibition in the bone marrow microenvironment by which stromal-derived factors such as FL and FGF2 mediate persistent activation of FLT3 and downstream signaling pathways potentially through activation of FLT3-WT.

Activation of parallel signaling pathways independent of the FLT3 receptor also plays a role in resistance to FLT3 inhibition. For example, upregulation of the receptor tyrosine kinase AXL has been implicated in resistance to TKIs in a number of tumor types [29–31]. Similarly, FLT3-ITD AML cells demonstrate constitutively active AXL and AXL inhibition leads to decreased FLT3 phosphorylation and induction of leukemia cell death [32]. Conversely, FLT3 inhibition leads to upregulation and activation of AXL and the degree of baseline AXL activity may predict sensitivity to the FLT3 inhibitor midostaurin. Moreover, AXL knockdown resensitized resistant FLT3-ITD cell lines to the effects of FLT3 inhibition, implicating AXL as a critical mediator of resistance [33]. AXL signals through many of the same downstream oncogenic pathways as FLT3 [34] so may serve as a bypass mechanism that allows leukemia cells to survive FLT3 inhibition.

The above findings highlight some of the challenges to successful translation of FLT3 inhibition to clinical application. A number of new inhibitors and dual inhibition strategies have been developed to overcome these barriers. Two of the newer generation FLT3 inhibitors that have advanced the furthest in clinical development are crenolanib and gilteritinib. Crenolanib is a type 1 TKI that is highly selective for FLT3 and PDGFR and retains a high level of activity against D835 mutant FLT3 and to a lesser degree against the F691 gatekeeper mutation [35,36]. In clinical trials single agent crenolanib has shown the best efficacy in FLT3 TKI naïve patients with 23% of patients achieving a complete remission with incomplete hematologic recovery (CRi); however, duration of response was again brief with a median time of 13 weeks [37]. Gilteritinib (ASP2215) is a newly developed dual FLT3/AXL inhibitor specifically designed to inhibit FLT3-ITD. In preclinical studies gilteritinib potently inhibited both ITD and activation loop mutations and have minimal activity against c-KIT and thus potential to limit the adverse myelosuppression seen with other inhibitors [38]. Additionally, as gilteritinib also inhibits AXL it is hypothesized to have improved anti-leukemic effects by preventing AXL activation as a this bypass mechanism; however, gilteritinib is even less potent against the gatekeeper mutation than crenolanib so patients would be still be susceptible to development of resistance by acquired mutations at this site. In a recently published phase 1/2 trial patients with mutated FLT3 (either ITD or activation loop) treated with gilteritinib monotherapy had a 41% composite complete remission with a mean duration of response of 20 weeks [39]. Further trials of crenolanib and gilteritinib alone and in combination with cytotoxic chemotherapy are ongoing and will be necessary to determine whether these new inhibitors are superior to the previous generation. Finally, we have developed MRX-2843, a novel small molecule dual inhibitor of MERTK and FLT3 [40]. MERTK and AXL are both members of the TAM family receptor tyrosine kinases and MERTK has been validated as a target in AML and other hematologic malignancies [41,42]. MRX-2843 has high potency for both MERTK and FLT3, is therapeutically effective in murine xenograft models with FLT3-ITD, D835, and F691 mutations [40], has received the Food and Drug Administration Investigational New Drug status and will be the first-in-class MERTK inhibitor used in clinical trials. Ongoing preclinical studies are aimed at determining whether MERTK plays a role similar to AXL in mediating resistance to FLT3 inhibition.

The ongoing struggles to translate the preclinical promise of FLT3 inhibition into successful clinical use underscore the importance of a deep understanding of the biology of target receptors when developing translational strategies. As it is unlikely that FLT3 inhibition alone will be sufficient to induce durable remissions, ongoing research should be aimed at devising rational combination strategies with other targeted agents based upon understanding of the molecular mechanisms at play. Other promising preclinical lines of investigation include combination with MEK inhibitors [24] or targeting of the bone marrow stromal interactions by CXCR4 inhibition [43,44]. Finally, most of the clinical trials to date have been performed in heavily pretreated adult patients with relapsed disease who are less likely to demonstrate any prolonged benefit from newly developed therapies and may have drastically altered drug metabolism as a confounding factor that diminishes the generalizability of the studies. Future studies should emphasize on the up-front use of FLT3 inhibitors in newly diagnosed and pediatric patients to maximize our understanding of their use in these critical populations.
